# Phage Therapy as Adjuvant to Conservative Surgery and Antibiotics to Salvage Patients With Relapsing *S. aureus* Prosthetic Knee Infection

**DOI:** 10.3389/fmed.2020.570572

**Published:** 2020-11-16

**Authors:** Tristan Ferry, Camille Kolenda, Cécile Batailler, Claude-Alexandre Gustave, Sébastien Lustig, Matthieu Malatray, Cindy Fevre, Jérôme Josse, Charlotte Petitjean, Christian Chidiac, Gilles Leboucher, Frédéric Laurent

**Affiliations:** ^1^Service des Maladies Infectieuses et Tropicales, Hôpital de la Croix-Rousse, Hospices Civils de Lyon, Lyon, France; ^2^Université Claude Bernard Lyon 1, Lyon, France; ^3^Centre interrégional de Référence pour la prise en charge des Infections Ostéo-Articulaires complexes (CRIOAc Lyon), Hospices Civils de Lyon, Lyon, France; ^4^CIRI – Centre International de Recherche en Infectiologie, Inserm U1111, Université Claude Bernard Lyon 1, CNRS, UMR5308, Ecole Normale Supérieure de Lyon, Univ Lyon, Lyon, France; ^5^Institut des Agents Infectieux, Laboratoire de bactériologie, Centre National de Référence des Staphylocoques, Hôpital de la Croix-Rousse, Hospices Civils de Lyon, Lyon, France; ^6^Service de Chirurgie Orthopédique, Hôpital de la Croix-Rousse, Hospices Civils de Lyon, Lyon, France; ^7^Pherecydes Pharma, Romainville, France; ^8^Service de Pharmacie Hospitalière, Hôpital de la Croix-Rousse, Hospices Civils de Lyon, Lyon, France

**Keywords:** bacteriophages, phage therapy, prosthetic-joint infection, *S. aureus*, phagotherapy

## Abstract

**Objectives:** To report the management of three consecutive patients with relapsing *Staphylococcus aureus* prosthetic knee infection (PKI) for whom explantation was not feasible who received a phage therapy during a “Debridement Antibiotics and Implant Retention” (DAIR) procedure followed by suppressive antimicrobial therapy.

**Methods:** Each case was discussed individually in our reference center and with the French National Agency (ANSM). The lytic activity of three phages targeting *S. aureus*, which was produced with a controlled and reproducible process, was assessed before surgery (phagogram). A hospital pharmacist extemporaneously assembled the phage cocktail (1 ml of 1 × 10^10^ PFU/ml for each phage) as “magistral” preparation (final dilution 1 × 10^9^ PFU/ml), which was administered by the surgeon directly into the joint, after the DAIR procedure and joint closure (PhagoDAIR procedure).

**Results:** Three elderly patients were treated with the PhagoDAIR procedure. Phagograms revealed a high susceptibility to at least two of the three phages. During surgery, all patients had poor local conditions including pus in contact to the implant. After a prolonged follow-up, mild discharge of synovial fluid persisted in two patients, for whom a subsequent DAIR was performed showing only mild synovial inflammation without bacterial persistence or super-infection. The outcome was finally favorable with a significant and impressive clinical improvement of the function.

**Conclusions:** The PhagoDAIR procedure has the potential to be used as salvage for patients with relapsing *S. aureus* PKI, in combination with suppressive antibiotics to avoid considerable loss of function. This report provides preliminary data supporting the setup of a prospective multicentric clinical trial.

## Introduction

Prosthetic-joint infection (PJI) is the most dramatic complication after joint arthroplasty. *Staphylococcus aureus* is frequently involved in patients with relapsing PJI as this bacterium is a strong biofilm producer, which facilitates its persistence on the implant surface (1). In patients with chronic PJI, the recommended strategy is prosthesis exchange to mechanically eradicate the biofilm (1–4). However, prosthesis explantation is sometimes not feasible, especially for the knee location in elderly patients with multiple comorbidities at risk of dramatic loss of function, reduction of the bone stock, fracture, or death. Debridement Antibiotics and Implant Retention (DAIR) could be used for such patients but the risk of relapse is particularly high due to the bacterial persistence in biofilm on the implant surface, even if suppressive antibiotic treatment (SAT) is usually proposed for these patients (1–4). In this context, the use of new adjuvant therapies that locally target the bacterial biofilm is of great interest as it may increase the success rate of SAT.

Lytic bacteriophages are viruses that specifically target bacteria (5). They are considered to have a high potential in patients with PJI, as it has been demonstrated that they have a synergistic anti-biofilm activity with antibiotics (6). In a patient with relapsing chronic PJI, we already performed DAIR and used bacteriophages that were injected into the joint with a good clinical response (7). Since then, three other consecutive patients included in the Lyon BJI cohort study (NCT02817711) and presenting a *S. aureus* relapsing prosthesis knee infection (PKI) in therapeutic dead-end (for whom revision was not feasible) benefited from DAIR with local administration of a cocktail of bacteriophages followed by SAT and were proposed in the present report.

## Methods

In accordance with the local ethics committee, each case was discussed individually during multidisciplinary meetings in our regional reference center (8), and then with the French National Agency for Medicines and Health Products Safety (ANSM) to validate that no other options could be proposed without excessive risk of loss of function or death. Each patient signed a written consent. Phages PP1493, PP1815, and PP1957 (from the Pherecydes Pharma library), targeting *S. aureus*, were produced with a controlled and reproducible process in an appropriate environment under the supervision of ANSM. These phages are strictly lytic natural phages isolated from environmental sources and selected for the complementarity of their host spectrum on a clinical reference panel of *S. aureus* (not shown). They belong to the *Silviavirus* and *Rosenblumvirus* genus (ICTV 2018). A “phagogram” was performed using two complementary techniques (spot plaque assay, kinetic assay) to assess the lytic activity of the bacteriophages on clinical strains collected from joint puncture performed before surgery (7). The DAIR procedure was performed during open surgery, as previously described (9). A hospital pharmacist extemporaneously assembled the cocktail of the three phages (1 ml of 1 × 10^10^ PFU/ml for each phage) as “magistral” preparation (final dilution 1 × 10^9^ PFU/ml), and each cocktail was administered by the surgeon directly into the joint, after the DAIR procedure and joint closure (PhagoDAIR procedure).

## Results

**Figure 1:**
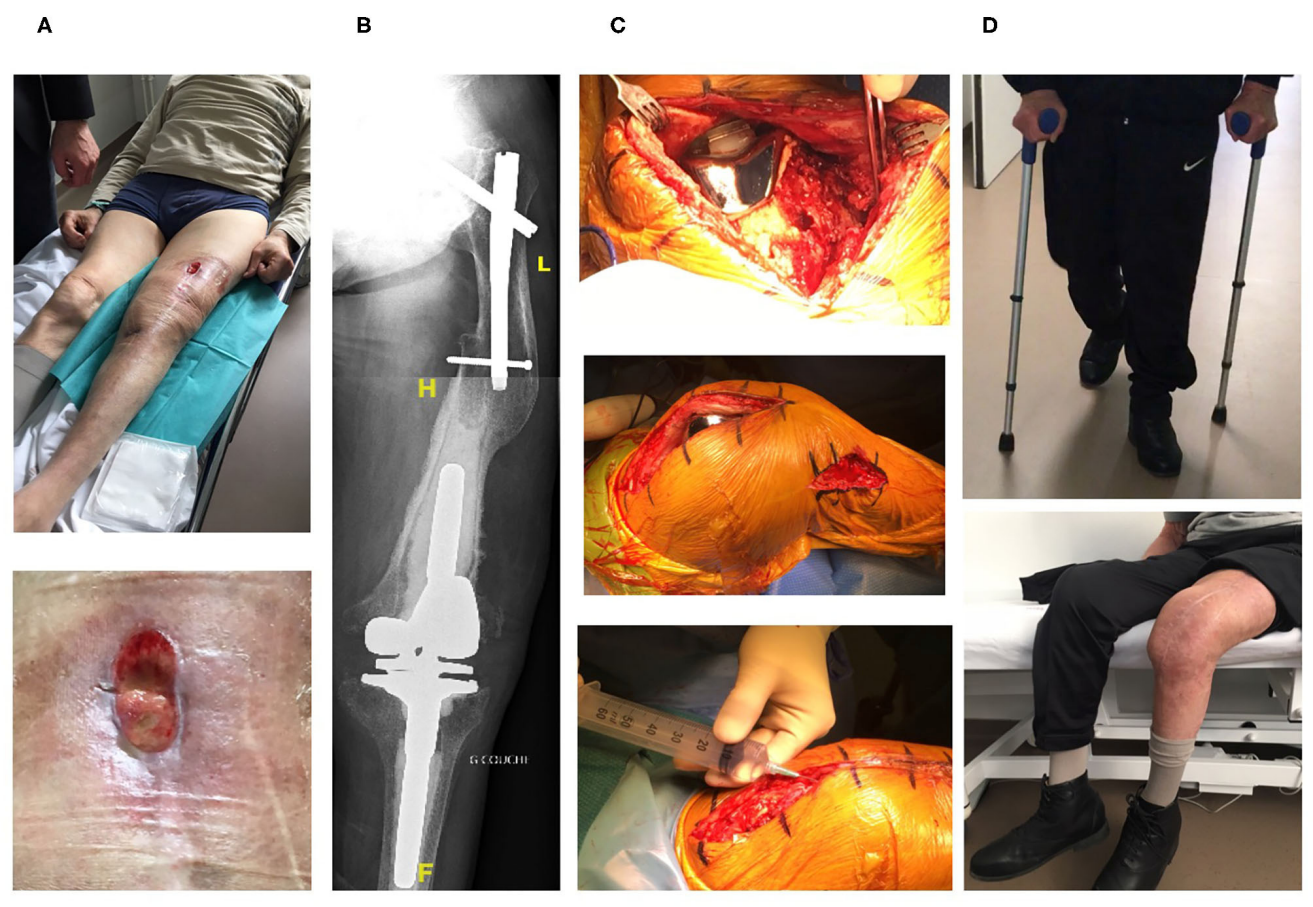
The first patient is an 80-year-old man with past history of Parkinson disease, cardiac arrhythmia, hypertension, right hip prosthetic joint, and left hip fracture treated with osteosynthesis. A left knee prosthetic joint arthroplasty was performed in 2004. In 2014, the patient developed signs of septic arthritis of the left knee due to a methicillin-susceptible *S. aureus*. A one-stage exchange was performed with reimplantation of a hinged knee prosthesis with long cemented stem. The patient received intravenous daptomycin and rifampin orally, followed by cotrimoxazole and clindamycin for a total duration of 3 months. Unfortunately, a relapse occurred in April 2015 with persistence of the *S. aureus*, and pristinamycin (a streptogramin A+B antibiotic available in France) was prescribed as suppressive antimicrobial therapy. Despite pristinamycin treatment, the patient developed signs of left septic arthritis with severe pain, bedridden and large anterior fistula with purulent discharge **(A)**. X-ray revealed a complex orthopedic situation with radiological signs of prosthesis loosening of the femoral stem **(B)**. Open DAIR was performed showing poor soft tissue condition with pus in contact to the implant and the personalized cocktail of phages was injected in joint just after closure **(C)**. The patient improved quickly, but at 3 months, a mild discharge of synoviual fluid persisted (Figure 5). A new DAIR was performed, revealing a drastic improvement of the local conditions, with only mild signs of non-specific synovitis. Multiple samples were performed for bacterial culture, but no recurrency/superinfection was diagnosed (cultures remained sterile, specific *S. aureus* PCR was negative). Cefalexin was then prescribed as suppressive therapy, and the outcome was favorable after 2 years and half of follow-up with no sign of infection, a negative C-reactive protein, and a pain-free walking [**(D)**, Supplementary Video 1].

**Figure 2:**
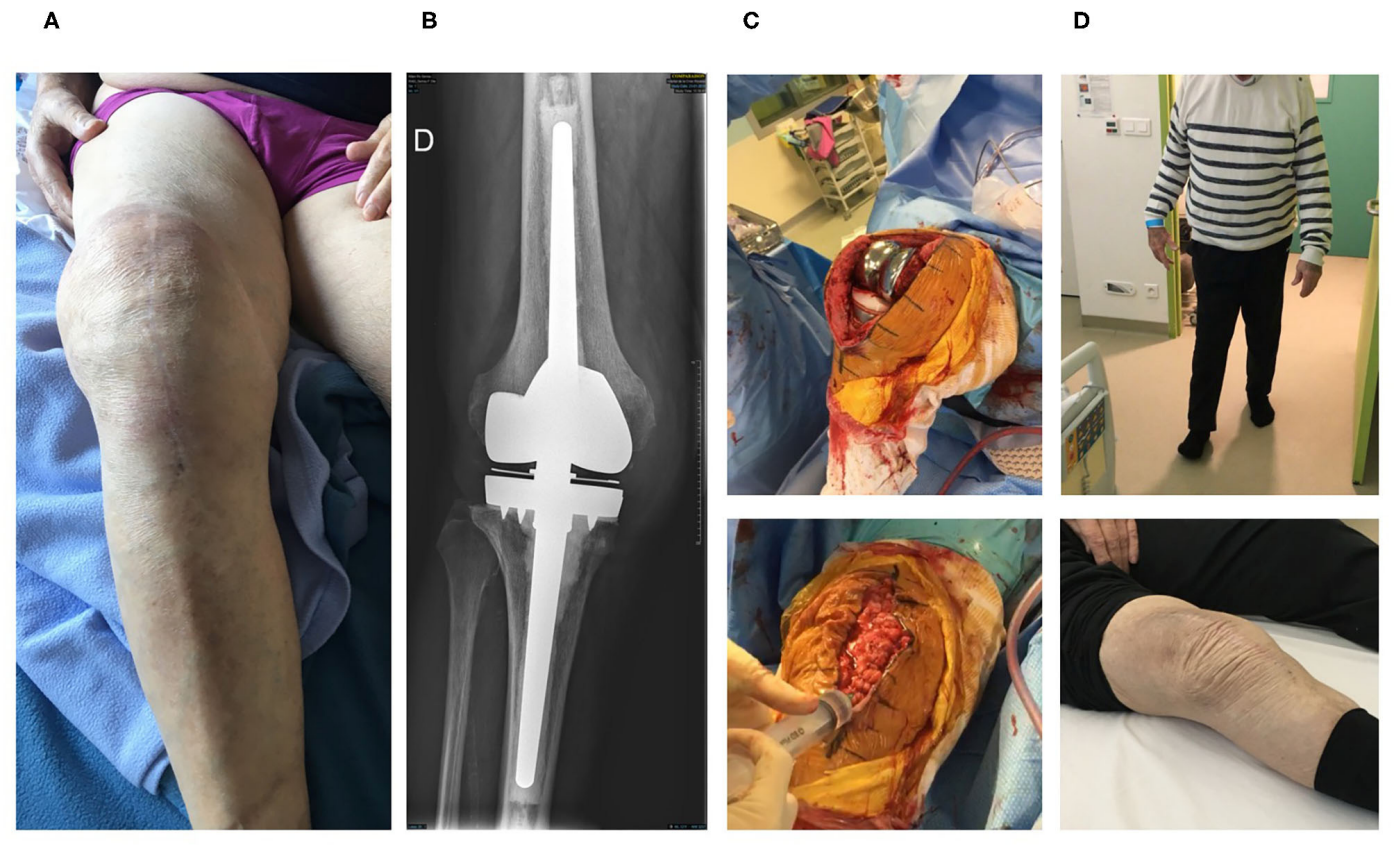
The second patient is an 84-year-old man with past history of dyslipidemia and right prosthetic-knee arthroplasty in 2006. A two-stage exchange was performed in 2007 for a *S. epidermidis* PKI. As the patient kept a painful knee, a one-stage exchange was performed in 2016, with implantation of a hinged cemented knee prosthesis with long stem. Cultures remained sterile. In 2019, the patient developed clinical signs of acute septic arthritis with fever. A DAIR without polyethylene exchange was performed and methicillin-susceptible *S. aureus* grew in perioperative samples and blood cultures. Endocarditis was excluded. The patient was treated using intravenous cefazolin and rifampin orally. Cefazolin was switched to ofloxacin 3 weeks after the DAIR and was combined with rifampin. Under this treatment, new signs of septic arthritis occurred 6 weeks after the DAIR, with local erythema, pain, and large joint effusion **(A)**. A joint puncture was performed, but no pathogen was isolated in cultures, and the failure was attributed to *S. aureus*. X-ray showed no loosening of the prosthesis **(B)**. Open DAIR was performed showing significant local inflammation and pus into the joint. The personalized cocktail of phages was administered after joint closure **(C)**. The patient improved quickly, doxycycline was then prescribed as suppressive therapy, and the outcome was favorable at 7 months with no signs of infection, a negative C-reactive protein and pain-free walking [**(D)**, Supplementary Video 2].

**Figure 3:**
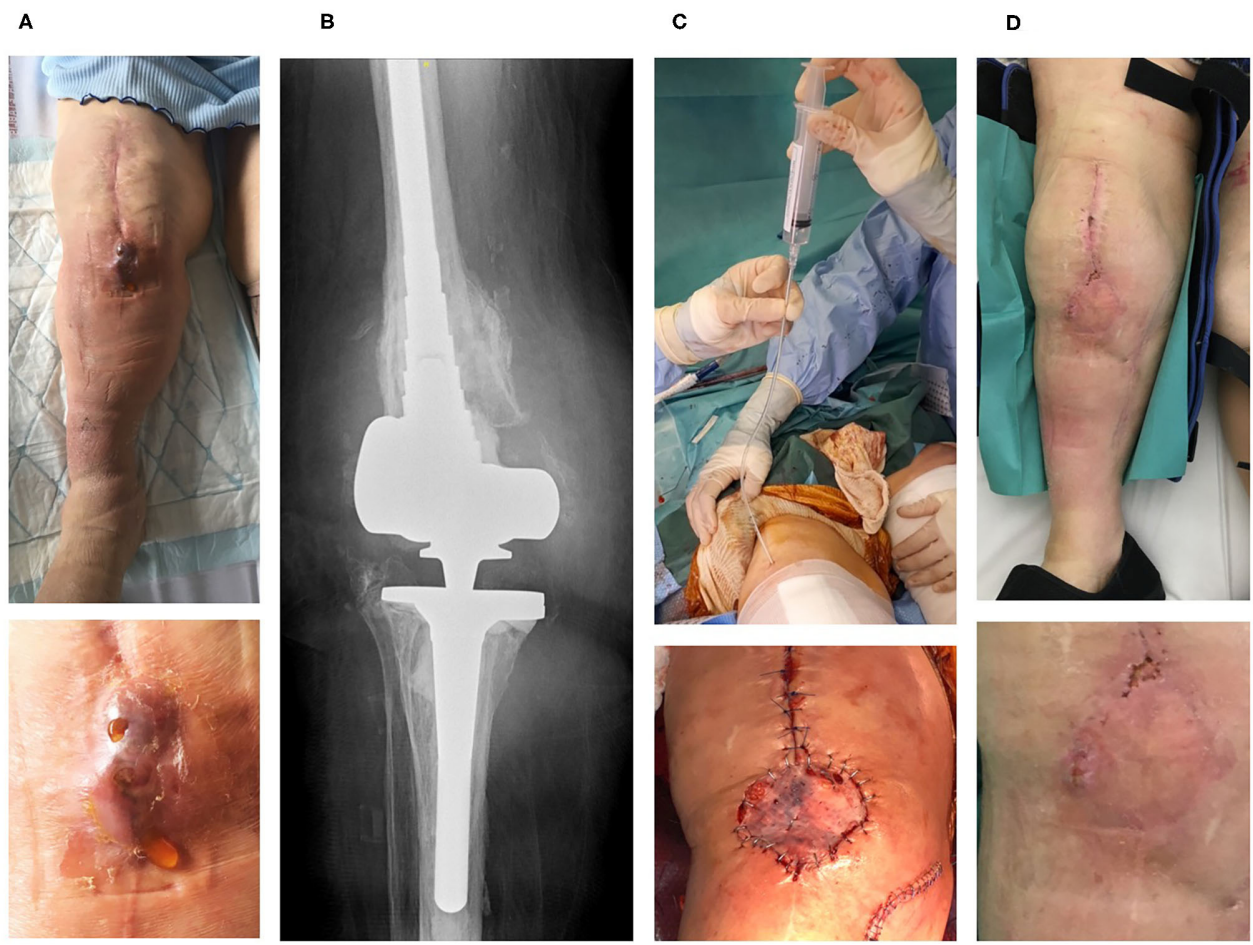
The third patient is an 83-year-old woman with past history of hypertension and lymphoedema. Right knee arthroplasty was performed in 2000. As prosthesis loosening occurred, a one-stage exchange was performed in 2018 with implantation of a hinged cemented knee prosthesis with long stem. In April 2019, a fistula occurred close to the tibial tuberosity, and in May 2019, the patient developed signs of PKI with fever. Open DAIR without polyethylene exchange was performed and methicillin-susceptible *S. aureus* grew in blood cultures, and in perioperative samples (with different phenotypes on agar for these latter, but with the same antibiogram. Determination of *agr* type by PCR showed that one strain belong to *agr* type I, and the other one to *agr* type II). Endocarditis was excluded. The patient received intravenous cloxacillin and rifampin orally. Unfortunately, the outcome was not favorable with occurrence of a large fistula with *Bourgeon charnu* **(A)**. A joint puncture was performed, but no pathogen was isolated in cultures, and the failure was attributed to *S. aureus*. X-ray did not reveal prosthesis loosening **(B)**. Open DAIR was performed showing catastrophic local condition with pus into the joint, and soft-tissue coverage with local flap was required. The personalized cocktail of phages was administered after joint closure, using a tube placed directly into the joint to preserve the flap **(C)**. The patient improved quickly, but a mild discharge of synovial fluid persisted after 4 months **(D)**. A new DAIR was performed, but no superinfection was diagnosed (cultures remained sterile, specific *S. aureus* PCR was negative). Doxycycline was then prescribed as suppressive therapy. At 11 months, a pain-free walking was observed, but the patient had persisting mild intermittent discharge of synovial fluid associated with a fistula and a C-reactive protein ≈20 mg/L (Figure 6, Supplementary Video 3), without any superinfection at the joint puncture performed at the end of the follow-up (cultures still sterile, and PCR still negative).

**Figure 4:**
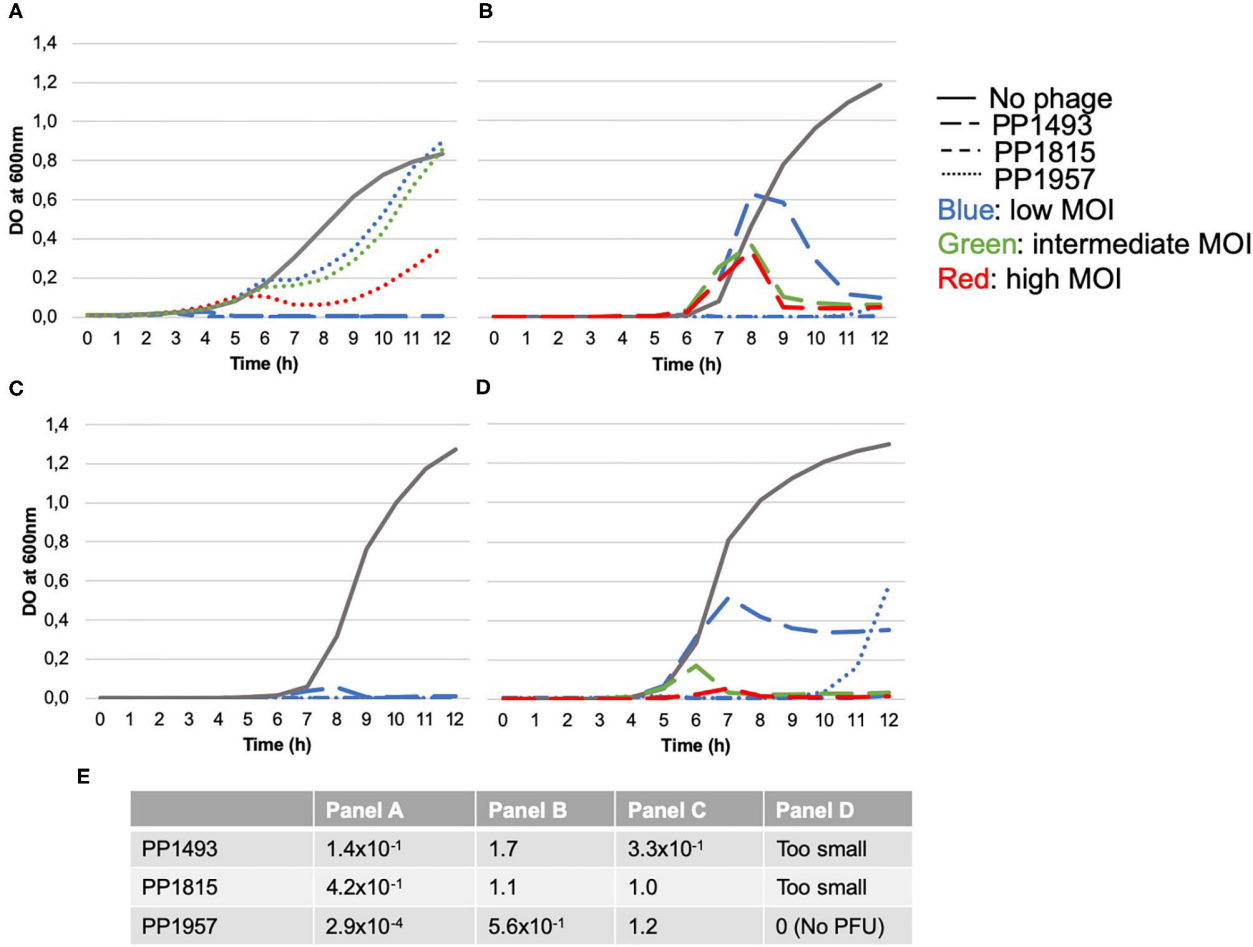
Phagograms performed using the kinetic assay **(A–D)** and the spot plaque assay **(E)**. For the kinetic assay, the bacterium was incubated with or without bacteriophages, tested individually at three different initial concentrations to obtain low, intermediate, and high multiplicity of infection (MOI, ratio of phages/bacteria). **(A)** Corresponds to bacterial growth kinetic (Optical Density at 600 nm) obtained for the strain isolated on patient 1; **(B)** from patient 2; and **(C,D)** from the two different strains isolated from patient 3. Except for PP1957 on strain A (that only delayed bacterial growth whatever the MOI), all phages were able to inhibit bacterial growth at high MOI **(A–D)**. Of note: (i) a slight delayed inhibition of the bacterial growth of strain B was observed with phage PP1493; (ii) only low MOI are represented concerning the phage activity on the strain C (blue lines), as intermediate and high MOI totally inhibited the bacterial growth; (iii) a partial growth inhibition of strain D (one of the two strains infecting patient 3) was observed with the phage PP1493 at low MOI, and a late growth of strain D appeared in presence of PP1957 at low MOI. The plaque test assay relies on the determination of the efficiency of plating score (EOP), calculated dividing the phage titer on the patient's strain by the phage titer on the reference strain (highly susceptible strain, used for phage amplification). The closer to 1 is the score is, the more efficient the phage is and likely active at low dose. Panel E showed the EOP scores of each strain and revealed that PP1493, PP1815, and PP1957 were active and very efficient on strains A, B, C from patients 1, 2, and 3 since EOP scores ranged from 1.4 × 10^−1^ to 1.7 with the exception of PP1957 strain A, which showed a low efficiency, consistently with kinetic assay results. However, for strain D (second strain from patient 3), PFU formed with PP1493 and PP1815 could be observed but they were too small to be enumerated confidently. The minimum concentration of spotted phages leading to PFU was 5.5 × 10^5^ and 3.9 × 10^4^ PFU/ml, respectively. Partial lysis without PFU was observed for PP1957 (result concordant with the kinetic assay as only a late growth at the lowest dose was observed).

**Figure 5:**
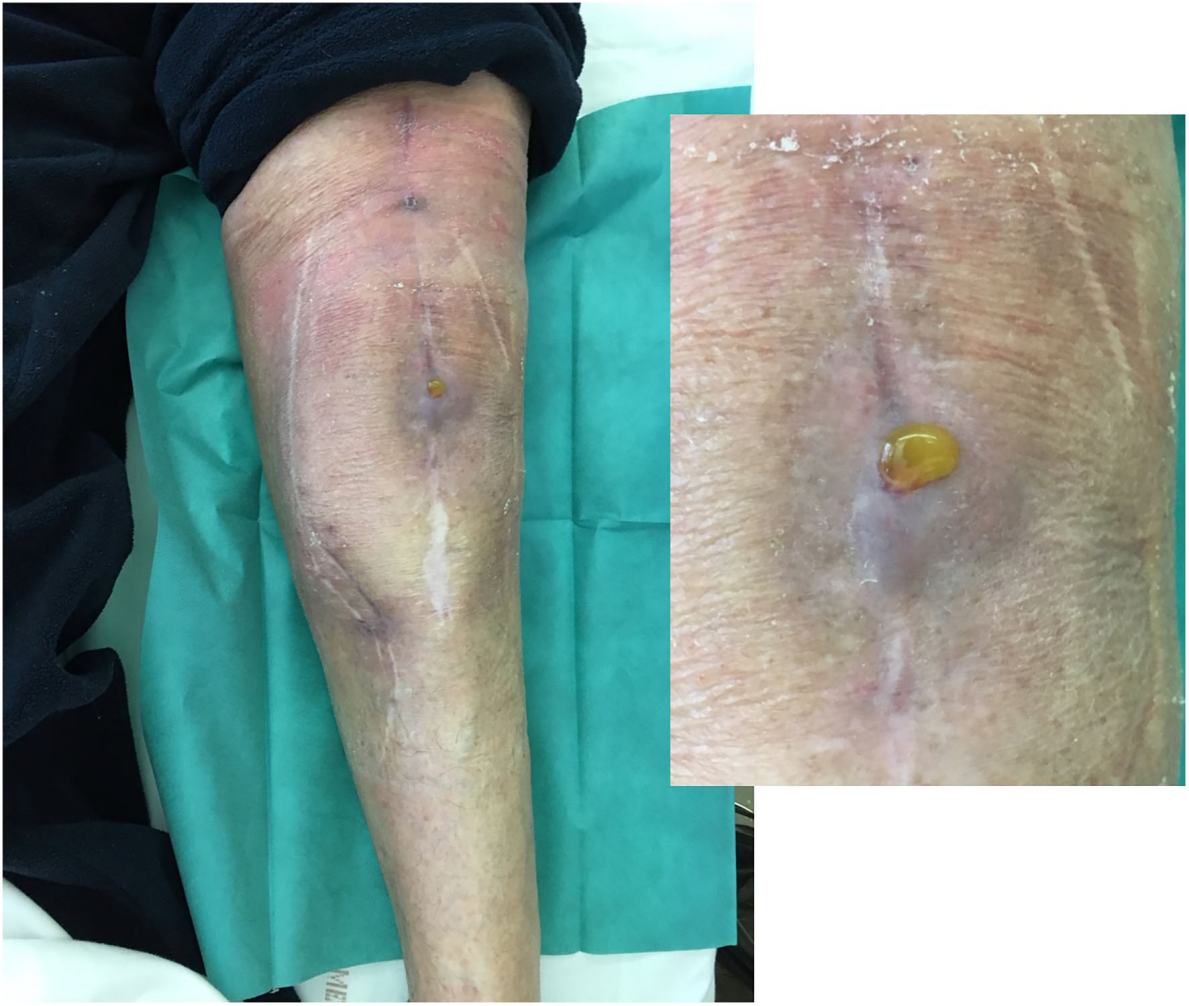
Local status of patient 1 showing, 3 months after the phagoDAIR procedure, the significant improvement of inflammatory signs of infection, with persistence of mild discharge of synovial fluid through the scar, for which a new DAIR was performed to exclude a superinfection. After the new DAIR, the outcome was favorable.

**Figure 6:**
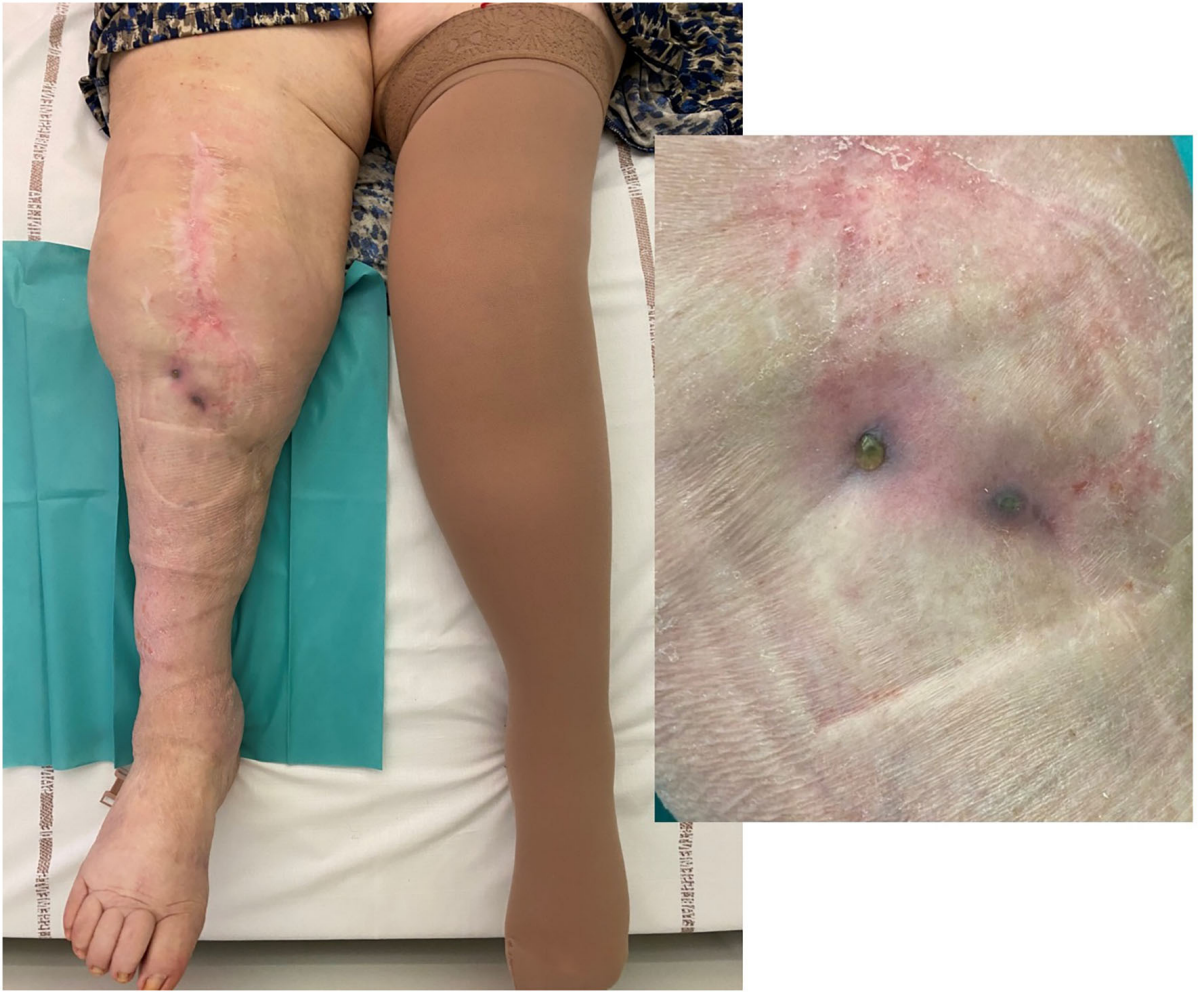
Local status of patient 3 (infected with two different *S. aureus* strains and for whom a soft-tissue coverage was required) showing 8 months after the phagoDAIR procedure a significant improvement of inflammatory signs of infection, but with fistula and discharge that persisted despite the phagoDAIR procedure.

Three consecutive elderly patients were treated with the PhagoDAIR procedure. All of them presented treatment failure despite a one-stage exchange followed by prolonged SAT (patient 1, Figure 1) or a previous DAIR followed by adequate antibiotics (patients 2 and 3; Figures 2, 3, respectively). All patients had knee prosthesis with long stem (revision prosthesis), without loosening, for whom polyethylene exchange was not feasible (Figures 1–3B). Phagograms revealed a high susceptibility to at least two of the three phages at high MOI (Figure 4). Phages with partial lytic activity, or phages only active at high MOI, were still preserved in the final cocktail, to prevent the acquisition of phage resistance under treatment, as these phenomena has been previously observed in a previous case report (10). Patient 3 was infected with two genetically different strains (*agr* typing), showing different phage susceptibility (Figures 4C,D). During surgery, all patients had poor local conditions including pus in contact to the implant (Figures 1–3C). Polyethylene exchange was technically not feasible, and soft tissue flap was required for one of them (Figure 3C). After the PhagoDAIR procedure, patients were treated with antibiotics in combination during 6 to 12 weeks, followed by SAT, according to the IDSA guidelines (Table 1) (2). After a follow-up of 7, 11, and 30 months, respectively, the outcome was favorable with a significant and impressive clinical improvement of the function for all patients (Figures 1–3D; Supplementary Videos 1–3). A mild discharge of synovial fluid persisted in two patients (patient 1, Figure 5), for whom a new DAIR was performed showing only mild synovial inflammation without bacterial persistence or super-infection. For these patients, a new phage administration was not performed. At the end of the follow-up, total disappearance of signs of infection was noticed except for one patient (patient 3, who was infected with two different *S. aureus* strains with different phage susceptibility) for whom a fistula with a mild intermittent synovial fluid discharge persisted despite the iterative DAIR (Figure 6).

**Table 1:** Details about the prosthetic knee infection history of the three patients treated with the PhagoDAIR procedure.

**Patient ID**	**Age (sex)**	**Putative mechanism of inoculation**	**Time since prosthesis implantation (months)**	**Duration of clinical symptoms before the PhagoDAIR procedure (days)**	**Delay from the previous surgery performed for the current infection to the PhagoDAIR procedure (days)**	**Antimicrobial resistance**	**Successive primary antimicrobial therapies after the PhagoDAIR procedure (duration in days)**	**Successive SAT after the primary antimicrobial therapy(ies) until the last follow-up (duration in days)**
Patient 1	80 (male)	Perioperative	40	976	One-stage exchange (1,371)	Penicillin G	Daptomycin–cloxacillin (4)n^*^ Levofloxacin–rifampin (123)	Doxycycline (45)^***^ Cephalexin (739)
Patient 2	84 (male)	Hematogenous	35	82	Open DAIR without PE exchange (78)	Erythromycin	Daptomycin–levofloxacin (14)^**^ Ofloxacin–doxycycline (72)	Doxycycline (189)
Patient 3	83 (female)	Perioperative	11	122	Open DAIR without PE exchange (98)	Penicillin G	Daptomycin–cefepime–rifampin (14)^**^ Levofloxacin–rifampin (111)	Doxycycline (200)

*SAT, suppressive antimicrobial therapy; DAIR, debridement antibiotics and implant retention; PE, polyethylene*.

* *This regimen was switched to oral antibiotics due to loss of the central line*.

** *This regimen was switched to oral antibiotics at the reception of the final culture results*.

*** *This regimen was switched to cephalexin due to oral ulceration attributed to doxycycline*.

## Discussion

We report here the impressive positive outcome of patients with relapsing *S. aureus* PKI treated with the PhagoDAIR procedure. This innovative procedure has been set up in our center for salvage therapy in patients with complex PJI after individual multidisciplinary and ethical discussions under supervision of ANSM. We previously published a case report using these phages, as salvage treatment, during a DAIR procedure in a patient with *S. aureus*, but also plurimicrobial, prosthetic hip infection. As we observed a positive outcome, we considered this approach as a possible opportunity to treat other patients with dead-end clinical situation (7). Of note, the three patients treated here experienced a previous treatment failure despite a one-stage exchange followed by prolonged SAT or despite a DAIR followed by adequate antibiotics. All of them had knee prosthesis with long stem (revision prosthesis), without loosening. For patient 1, the previous medico-surgical treatment was optimal, with a one-stage exchange followed by adequate antimicrobial antibiotics during 3 months. However, pristinamycin (a streptogramin A+B antibiotic available in France) was directly prescribed 1 year later, at the diagnosis of relapse, without any subsequent surgery. For patient 2, DAIR and adequate antibiotics were prescribed for a hematogenous infection, but polyethylene was not exchanged during DAIR, and the relapse occurred during rifampin–ofloxacin treatment. Concerning patient 3, a DAIR procedure and adequate antibiotics potentially followed by SAT were proposed, even if the infection was chronic. This later patient also experience a failure under antimicrobial therapy that included rifampin.

Globally, in patients with PJI, targeting the biofilm is a potential key determinant. In patients with chronic infection, if getting rid of the biofilm by prosthesis exchange is not feasible, DAIR followed by SAT is usually proposed, but the success rate remains low (1–4). By using phage therapy as adjunctive therapy, the aim is to act locally on bacteria embedded in biofilm stuck on the implant surface into the joint cavity, as demonstrated recently in an animal model (11). The anti-*S. aureus* phages used to treat our patients demonstrated dose-dependent anti-biofilm activity *in vitro*. In addition, in the same study, synergistic effects were reported when phages were combined with antibiotics used at concentrations below MICs (6).

This report has several major limitations: (i) the non-comparative design, (ii) the small number of patients, (iii) the use of phage therapy as adjuvant to surgery and antibiotics that leads to question about the intrinsic capacity of the phage therapy to improve the outcome, and (iv) the subsequent DAIR performed in two patients during the follow-up. However, the clinical history of the three patients was homogeneous, with a dead-end situation. As they presented relapsing *S. aureus* PKI after previous standard of care treatments, the expected success rate of iterative DAIR procedure followed by SAT was close to zero. First of all, *S. aureus per se* is considered as the most virulent pathogen in PJI and is an independent risk for DAIR failure (9). Secondly, a previous one-stage or DAIR procedure was performed, unsuccessfully, and a subsequent DAIR is an independent risk factor for failure (12). Finally, it was technically not feasible to replace the polyethylene in these patients, which has been associated with failure in several studies (2–4, 12). Concerning the DAIR performed in two patients after the PhagoDAIR procedure, the indication was based on the persistence of a mild discharge of synovial fluid, whereas all patients had already improved significantly, and only non-specific mild synovitis, without positive cultures, was found. For one of these patients, the outcome was finally favorable. Put together, these different points suggest that the PhagoDAIR procedure highly participated into the clinical improvement in the patients reported here.

## Conclusion

The PhagoDAIR procedure has the potential to be used as salvage for patients with relapsing *S. aureus* PKI, in combination with suppressive antibiotics to avoid considerable loss of function. This report provides preliminary data supporting the setup of a prospective multicentric clinical trial.

## Data Availability Statement

The raw data supporting the conclusions of this article will be made available by the authors, without undue reservation.

## Ethics Statement

The studies involving human participants were reviewed and approved by Hospices Civils De Lyon Ethic Committee. Written informed consent to participate in this study was provided by the participants' legal guardian/next of kin. Written informed consent was obtained from the individual(s), and minor(s)' legal guardian/next of kin, for the publication of any potentially identifiable images or data included in this article.

## Author Contributions

TF managed all the patients, directly interacted with the French Health authority, and wrote the manuscript. CB, SL, and MM participated to the surgical management. CK, C-AG, CF, JJ, and CP performed bacteriological experiments. GL performed the magistral preparation. All authors participated in the literature review and the improvement of the manuscript.

## Conflict of Interest

CF and CP are employed by the company Pherecydes Pharma. The remaining authors declare that the research was conducted in the absence of any commercial or financial relationships that could be construed as a potential conflict of interest.

## Supplementary Material

Supplementary Video 1 Video showing patient 1 walking without any pain 1 year after the PhagoDAIR procedure.

Supplementary Video 2 Video showing patient 2 walking without any pain 7 months after the PhagoDAIR procedure.

Supplementary Video 3 Video showing patient 2 walking without any pain 11 months after the PhagoDAIR procedure.

## References

[1] Zimmerli W Trampuz A Ochsner PE. Prosthetic-joint infections. N Engl J Med. (2004) 351:1645–54. 10.1056/NEJMra040181 15483283

[2] Osmon DR Berbari EF Berendt AR Lew D Zimmerli W Steckelberg JM Diagnosis and management of prosthetic joint infection: clinical practice guidelines by the infectious diseases society of America. Clin Infect Dis. (2013) 56:e1–25. 10.1093/cid/cis803 23223583

[3] Société de Pathologie Infectieuse de Langue Française (SPILF) Collège des Universitaires de Maladies Infectieuses et Tropicales (CMIT) Groupe de Pathologie Infectieuse Pédiatrique (GPIP) Société Française d'Anesthésie et de Réanimation (SFAR) Société Française de Chirurgie Orthopédique et Traumatologique (SOFCOT) Société Française d'Hygiène Hospitalière (SFHH) Société Française de Médecine Nucléaire (SFMN) Recommendations for bone and joint prosthetic device infections in clinical practice (prosthesis, implants, osteosynthesis). société de pathologie infectieuse de langue française. Med Mal Infect. (2010) 40:185–211. 10.1016/j.medmal.2009.12.009 20303685

[4] Ariza J Cobo J Baraia-Etxaburu J Benito N Bori G Cabo J. Executive summary of management of prosthetic joint infections. clinical practice guidelines by the Spanish society of infectious diseases and clinical microbiology (SEIMC). Enferm Infect Microbiol Clin. (2017) 35:189–95. 10.1016/j.eimce.2017.02.013 28215487

[5] Ferry T Boucher F Fevre C Perpoint T Chateau J Petitjean C. Innovations for the treatment of a complex bone and joint infection due to XDR *Pseudomonas aeruginosa* including local application of a selected cocktail of bacteriophages. J Antimicrob Chemother. (2018) 73:2901–3. 10.1093/jac/dky263 30060002

[6] Kolenda C Josse J Medina M Fevre C Lustig S Ferry T Evaluation of the activity of a combination of three bacteriophages alone or in association with antibiotics on *Staphylococcus aureus* embedded in biofilm or internalized in osteoblasts. Antimicrob Agents Chemother. (2020) 64:e02231–14. 10.1128/AAC.02231-19 31871084 PMC7038305

[7] Ferry T Leboucher G Fevre C Herry Y Conrad A Josse J. Salvage Debridement, Antibiotics and Implant Retention (“DAIR”) with local injection of a selected cocktail of bacteriophages: is it an option for an elderly patient with relapsing Staphylococcus aureus prosthetic-joint infection? Open Forum Infect Dis. (2018) 5:ofy269 10.1093/ofid/ofy269 30474047 PMC6240628

[8] Ferry T Seng P Mainard D Jenny JY Laurent F Senneville E. The CRIOAc healthcare network in France: a nationwide health ministry program to improve the management of bone and joint infection. Orthop Traumatol Surg Res. (2019) 105:185–90. 10.1016/j.otsr.2018.09.016 30413338

[9] Byren I Bejon P Atkins BL Angus B Masters S McLardy-Smith P. One hundred and twelve infected arthroplasties treated with “DAIR” (debridement, antibiotics and implant retention): antibiotic duration and outcome. J Antimicrob Chemother. (2009) 63:1264–71. 10.1093/jac/dkp107 19336454 PMC2680346

[10] Schooley RT Biswas B Gill JJ Hernandez-Morales A Lancaster J Lessor L. Development and use of personalized bacteriophage-based therapeutic cocktails to treat a patient with a disseminated resistant acinetobacter baumannii infection. Antimicrob Agents Chemother. (2017) 61:e00954–17. 10.1128/AAC.00954-17 28807909 PMC5610518

[11] Morris JL Letson HL Elliott L Grant AL Wilkinson M Hazratwala K Evaluation of bacteriophage as an adjunct therapy for treatment of peri-prosthetic joint infection caused by *Staphylococcus aureus*. PLoS ONE. (2019) 14:e0226574 10.1371/journal.pone.0226574 31877146 PMC6932802

[12] Lora-Tamayo J Murillo O Iribarren JA Soriano A Sánchez-Somolinos M Baraia-Etxaburu JM A large multicenter study of methicillin-susceptible and methicillin-resistant Staphylococcus aureus prosthetic joint infections managed with implant retention. Clin Infect Dis. (2013) 56:182–94. 10.1093/cid/cis746 22942204

